# Ocean Acidification Accelerates Reef Bioerosion

**DOI:** 10.1371/journal.pone.0045124

**Published:** 2012-09-18

**Authors:** Max Wisshak, Christine H. L. Schönberg, Armin Form, André Freiwald

**Affiliations:** 1 Marine Research Department, SENCKENBERG am Meer, Wilhelmshaven, Germany; 2 Oceans Institute at the University of Western Australia, Australian Institute of Marine Science (AIMS), Crawley, Western Australia, Australia; 3 Marine Biogeochemistry, GEOMAR - Helmholtz Centre for Ocean Research, Kiel, Germany; University of Gothenburg, Sweden

## Abstract

In the recent discussion how biotic systems may react to ocean acidification caused by the rapid rise in carbon dioxide partial pressure (*p*CO_2_) in the marine realm, substantial research is devoted to calcifiers such as stony corals. The antagonistic process – biologically induced carbonate dissolution via bioerosion – has largely been neglected. Unlike skeletal growth, we expect bioerosion by chemical means to be facilitated in a high-CO_2_ world. This study focuses on one of the most detrimental bioeroders, the sponge *Cliona orientalis*, which attacks and kills live corals on Australia’s Great Barrier Reef. Experimental exposure to lowered and elevated levels of *p*CO_2_ confirms a significant enforcement of the sponges’ bioerosion capacity with increasing *p*CO_2_ under more acidic conditions. Considering the substantial contribution of sponges to carbonate bioerosion, this finding implies that tropical reef ecosystems are facing the combined effects of weakened coral calcification and accelerated bioerosion, resulting in critical pressure on the dynamic balance between biogenic carbonate build-up and degradation.

## Introduction

Since the turn of the millennium, ocean acidification (OA) has been recognized as a key factor in marine ecology, attracting a growing pool of research which identified OA to have a multitude of mainly negative effects on reproduction, growth, survival, and diversity of marine biota [1]–[3]. Among the best studied victims in this respect are organisms that produce carbonate skeletons, and particularly scleractinian corals that show significantly reduced skeletal growth rates with declining pH and lowered seawater carbonate saturation state [4]–[8]. In contrast, bioeroding organisms have largely been ignored, although they play a key role in carbonate cycling by abrading or dissolving materials such as coral skeletons, and thus need to be included in any equation concerning reef health or growth. This omission needs to be addressed, because chemically achieved bioerosion is expected to be facilitated with progressing OA [9], [10], potentially placing many bioeroders into the circle of “winners” of global climate change [11].

**Figure 1:**
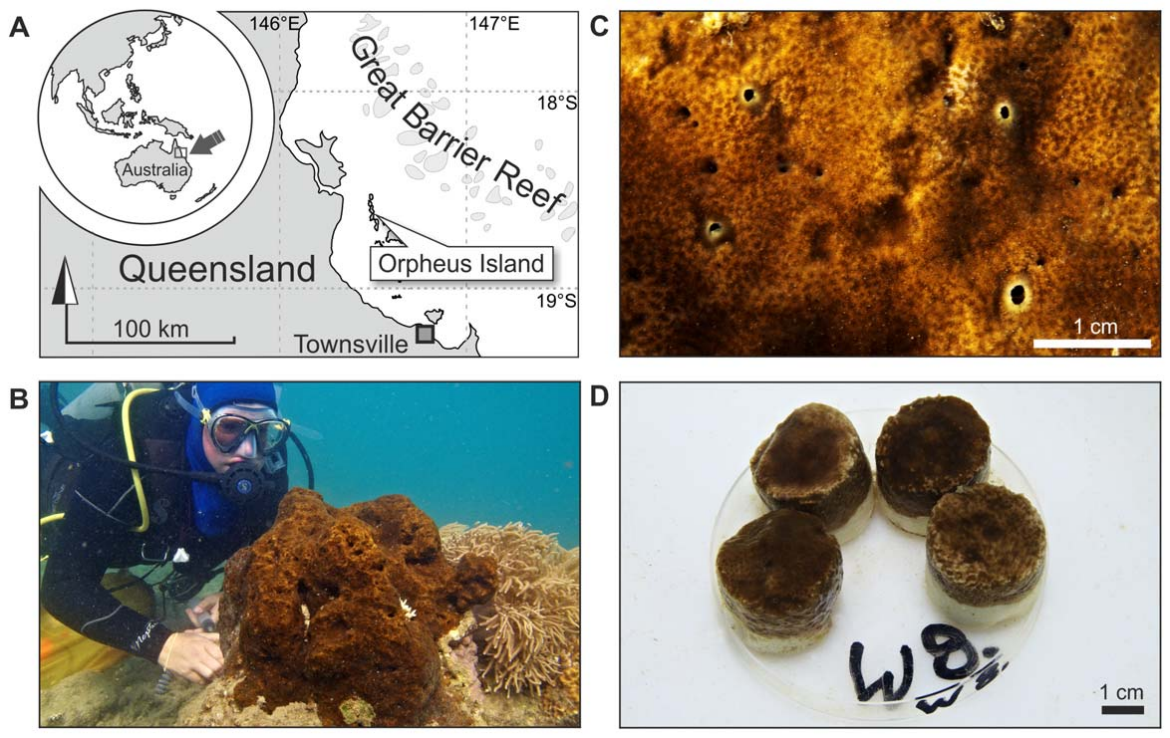
The zooxanthellate sponge *Cliona orientalis* at Orpheus Island, Great Barrier Reef, Australia. (A) Location of Orpheus Island (Palm Island Group) on the central GBR. (B) Medium-sized colony infesting the massive coral *Porites* sp. at the reef crest in Little Pioneer Bay, Orpheus Island. (C) Detail illustrating the oscula (exhalant pores; inhalant pores are microscopically small) and the *Porites* skeletal structure visible beneath the sponge tissue. (D) One of the eight replicate sets per treatment tank with 4 healed sponge-bearing coral cores.

Marine bioerosion acts at different scales and is performed by a multitude of organisms employing different chemical and mechanical means in the process of attachment, grazing, or carbonate penetration [12]. On coral reefs, the largest proportion of internal bioerosion is often contributed by demosponges, which do not add to calcification as they have siliceous spicules, but frequently represent 60 to over 90% of total macroborer activity [13], [14]. Single sponge species commonly remove around 10 and in extreme cases more than 20 kg m^−2^ yr^−1^ [15], thereby balancing or even surpassing reef calcification rates at some sites [14], [16]–[17]. In warm waters worldwide, the photosymbiotic clionaids of the so-called ‘*Cliona viridis* species complex’ lead this process in terms of abundance, colony size, growth, and erosion rates [15], [18]. Their symbiosis with dinoflagellate zooxanthellae appears to increase their competitive powers, and it is comparatively stress resistant [19], [20]. ‘*C. viridis* species’ routinely invade and kill live corals and have been reported to survive and increase in abundance where environmental conditions deteriorate [21]. Our model organism *Cliona orientalis* Thiele, 1900 belongs to this species complex and is one of the most competitive and abundant representatives of these bioeroders. It is widely distributed on Australia’s Great Barrier Reef (GBR), Indonesia and Japan [18], [22] (Fig. 1).

Sponges erode at cellular level by means of biochemical dissolution that leads to the formation of minute cup-shaped grooves and the mechanical extraction of so-called sponge chips of a diameter between 10 and 100 µm [22]. In order to dissolve carbonate, the sponge lowers the pH at the tissue-substrate interface where the specialised etching cells act [22] (exact etching agent unknown to date). Sponge bioerosion is conducted extracellulary potentially making the process sensitive to environmental conditions and change. The lower the environmental pH is to begin with, the less pronounced is the gradient between ambient seawater and the site of dissolution, and the lower will be the metabolic cost required for bioerosion. Hence we hypothesise that the pH lowering inherent to OA will increase the efficiency of the bioerosion process, leading to a significant increase of sponge bioerosion rates with increasing *p*CO_2_.

## Materials and Methods

### Experimental Setup

**Figure 2:**
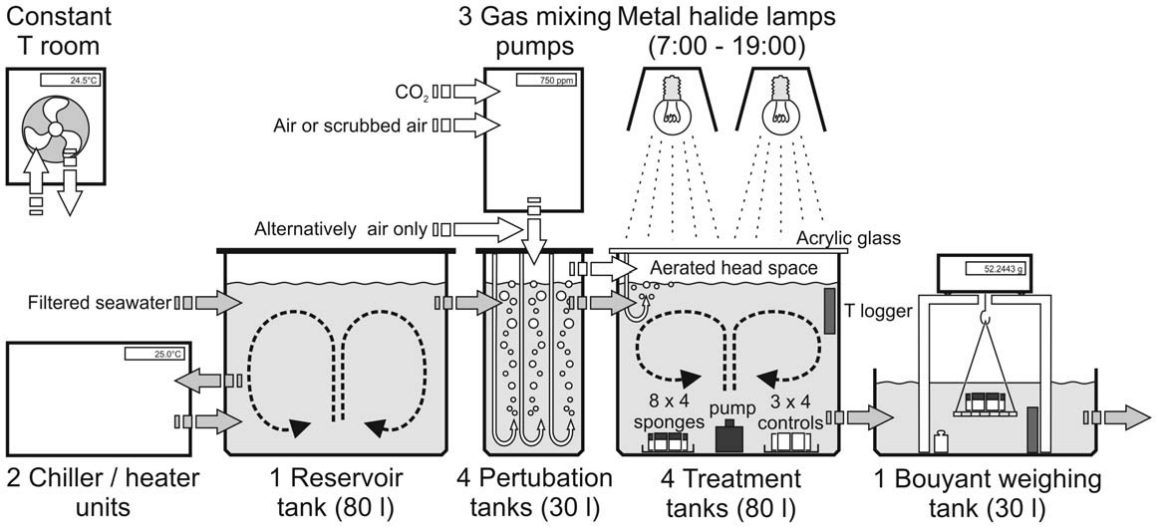
The experimental setup. Low-flow open system in a constant temperature room (T = 25°C) using filtered sea-water (25 µm) stored in a reservoir tank, with four treatment lines (*p*CO_2_ = 339 µatm, 393 µatm, 571 µatm, and 1410 µatm) each comprising a perturbation tank connected to a gas mixing pump, leading to an illuminated (12∶12 h) treatment tank with replicate petri dishes (n = 8 per treatment, containing 4 sponge-bearing coral cores) and controls (n = 3 per treatment, containing 4 clean coral cores), terminating in the buoyant weighing unit.

**Figure 3:**
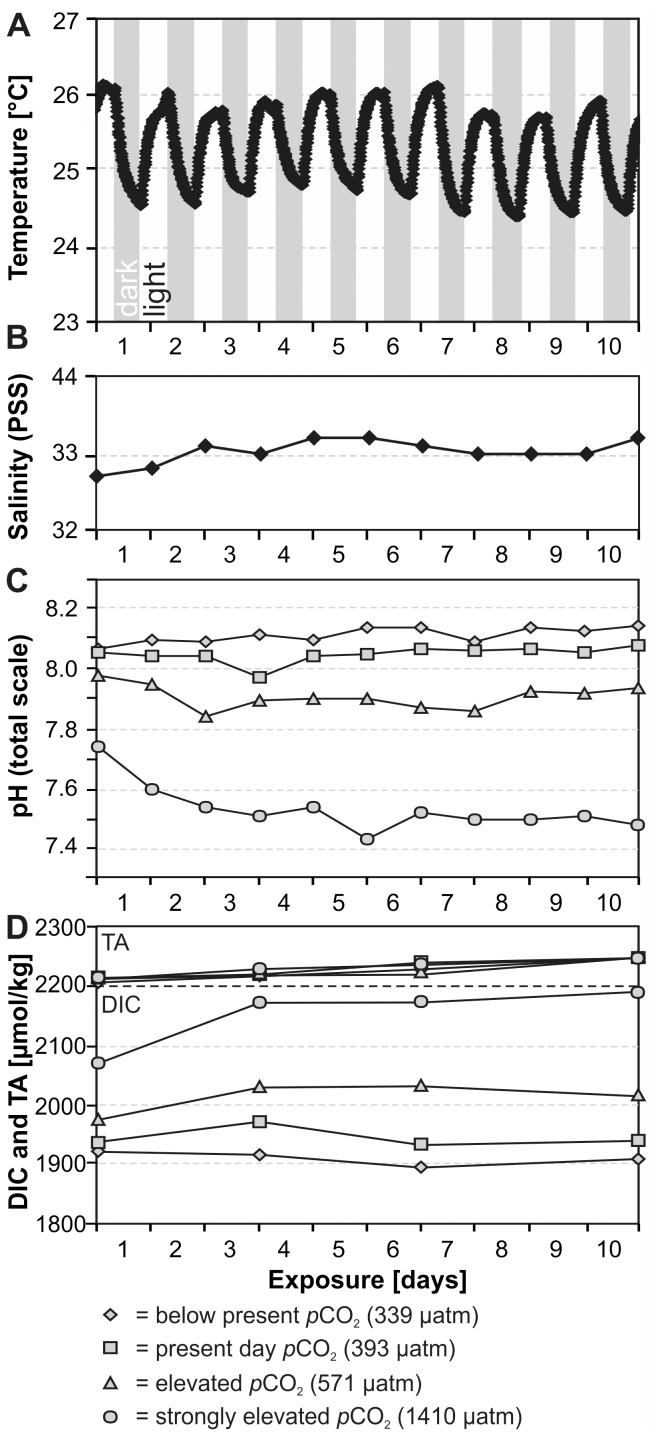
Temporal variability of measured experimental settings and carbonate system parameters during the 10 day experiment. (A) Diurnal temperature oscillation as a function of heat radiation during the illumination phases; identical for all four treatments. (B) Salinity of the incoming seawater; identical for all four treatments. (C) Measured pH levels; for monitoring purposes only. (D) TA (top) and DIC (bottom).

In order to test the physiological response in the bioerosion capacity of *Cliona orientalis* to simulated OA, we core-sampled sponge tissue with dead coral substrate from infested, but live, massive *Porites* sp. colonies in Little Pioneer Bay at Orpheus Island, central GBR (Fig. 1A). Sampling was achieved with an air-drill and hole-saw (inner diameter 30 mm), cores were trimmed to 25 mm in length with an air-cutter, and kept 4 days outdoors in a seawater flow-through raceway for recovery allowing the tissue to fully heal (Fig. 1D). Cores included sponge-penetrated material at the surface and clean dead coral skeleton below. Entirely clean cut-off parts of cores from the same colonies were prepared as controls and treated in exactly the same way. The condition of the physiologically important photosymbiosis with intracellular dinoflagellates was monitored with pulse amplitude modulated fluorescence (PAM) [20] by measuring their photosynthetic efficiency Fv/Fm and the proxy for chlorophyll *a* concentration F0 at the surface of dark-adapted sponge cores before and after the experiment (Maxi-iPAM, Walz, Germany). This analysis excluded significant differences between treatments and only in case of F0 it showed a moderate decrease over the course of the experiment, probably due to a partial retraction of the photosymbionts as a reaction to the experimental conditions (Table 1). Cores were transferred to a flow-through open system (Fig. 2) that was set up in a constant-temperature room (25°C), allowing 24 h of acclimatisation. Incoming ambient seawater taken from few hundred metres south of the sampling site in Pioneer Bay, was filtered to 25 µm, thereby retaining pico- and nanoplankton (<20 µm) as the sponges’ natural food. The water was temperature-adjusted in a reservoir tank coupled to two chiller/heater units (TC 15, Teco, Italy). Four outlets delivered controlled, constant flow (∼30 l/h) to the four treatment lines. The range of target *p*CO_2_ levels and the respective carbonate system parameters dissolved inorganic carbon (DIC), pH, and the resulting saturation state for aragonite and calcite in seawater (Ω_Ar_ and Ω_Ca_), were established via perturbation with specifically mixed gases in sealed 30 l tanks (Table 1, Figs. 2–3). This approach is accepted as an effective and the most appropriate method for simulating future carbonate system scenarios in closed, and particularly in open experimental systems [23]. The present-day level (*p*CO_2_ = 393 µatm; pH_(total scale)_ = 8.05) was provided in form of compressed air. Gases for the three manipulated *p*CO_2_ treatments were mixed with Digamix 5KA 36A/9 pumps (H. Wösthoff, Germany). The below present level (*p*CO_2_ = 339 µatm; pH_(total scale)_ = 8.10) was mixed from ‘scrubbed’ air, generated via CO_2_-assimilation by soda lime pellets (DiveSorb Pro, Dräger, Germany), and CO_2_. The elevated (*p*CO_2_ = 571 µatm; pH_(total scale)_ = 7.91) and the strongly elevated treatment levels (*p*CO_2_ = 1410 µatm; pH_(total scale)_ = 7.57) were mixed using food-grade CO_2_ and compressed air with ambient *p*CO_2_. The gas-adjusted seawater was led into 80 l treatment tanks. These were covered with lids of transparent acrylic glass to minimise evaporation and to stabilise the *p*CO_2_ in the headspace that was also filled with the respective gas mixture. Each flow-through treatment tank carried 8 replicate sets of 4 sponge-bearing cores taken from different coral colonies (Fig. 1D; 32 cores in total per treatment tank), and 3 sets of control cores (12 cores per treatment). In each tank, current was generated by a pump placed centrally, spout pointing upwards. The experiment ran for 10 days.

**Table 1:** Summary of experimental settings, carbonate system parameters, nutrient levels, photosynthesis parameters, and sponge bioerosion figures (all values are given as mean values ± standard deviation for 10 days of exposure).

Variable with [unit] or (scale)	below present *p*CO_2_	present-day *p*CO_2_	elevated *p*CO_2_	strongly elevated *p*CO_2_
temperature [°C]	25.27±0.51	25.27±0.51	25.27±0.51	25.27±0.51
salinity (PSS)	33.15±0.23	33.14±0.24	33.14±0.24	33.15±0.23
pH (NBS scale)	8.24±0.02	8.17±0.03	8.02±0.04	7.63±0.08
pH (converted to total scale)	8.11±0.02	8.04±0.03	7.90±0.03	7.55±0.07
DIC [µmol/kg]	1911.7±11.3	1945.4±19.7	2011.6±27.7	2153.3±55.7
TA [µmol/kg]	2226.1±16.6	2231.0±13.8	2225.7±17.6	2232.3±14.5
*p*CO_2_ [µatm]*	338.6±37.4	393.2±55.6	570.9±82.4	1409.5±369.6
pH (total scale)*	8.10±0.04	8.05±0.05	7.91±0.05	7.57±0.12
HCO_3_^−^ [µmol/kg]*	1679.6±26.3	1730.2±35.7	1836.8±39.2	2031.4±67.4
CO_3_^2−^ [µmol/kg]*	222.6±16.9	204.2±18.5	158.7±16.1	82.2±22.3
Ω_Ar_ *	3.59±0.27	3.29±0.29	2.56±0.26	1.32±0.36
Ω_Ca_ *	5.45±0.40	5.00±0.45	3.89±0.40	2.01±0.55
nitrate NO_3_ [µmol/l]	0.05±0.05	0.03±0.02	0.02±0.01	0.03±0.05
nitrite NO_2_ [µmol/l]	0.04±0.01	0.03±0.01	0.04±0.02	0.03±0.00
ammonium NH_4_ [µmol/l]	0.14±0.23	0.10±0.15	0.04±0.04**	0.05±0.03
phosphate PO_4_ [µmol/l]	0.08±0.02	0.08±0.02	0.07±0.03	0.07±0.02
silicate SiO_2_ [µmol/l]	4.80±0.41	4.85±0.49	4.69±0.60	4.89±0.44
luminous intensity at start [cd]	7359.4±343.5	7078.0±752.8	6782.1±415.7	6898.5±422.6
Fv/Fm before treatment	0.68±0.01	0.68±0.01	0.68±0.00	0.68±0.00
Fv/Fm after treatment	0.60±0.01	0.59±0.01	0.61±0.01	0.61±0.01
F0 before treatment	0.22±0.01	0.22±0.01	0.22±0.01	0.22±0.01
F0 after treatment	0.13±0.02	0.14±0.01	0.13±0.01	0.14±0.01
penetration depth [cm]	1.25±0.13	1.30±0.20	1.37±0.12	1.34±0.09
sponge biomass [g]	1.49±0.16	1.50±0.07	1.54±0.08	1.48±0.06
weight change per replicate [g]	−0.32±0.07	−0.32±0.02	−0.38±0.04	−0.52±0.06
change relative to present-day [%]	99.76±19.98		116.91±11.04	161.11±18.11
weight change per control [g]	0.08±0.02	0.05±0.00	0.05±0.02	0.04±0.01
bioerosion rate [kg m^−2 ^yr^−1^]	2.22±0.45	2.23±0.15	2.60±0.25	3.59±0.40

* Carbonate system parameters computed with the software CO2SYS.

** One contaminated sample excluded from analysis.

### Simulating a Diurnal Rhythm

The sponge-bearing cores were kept in a 12/12 h light/dark rhythm. This was achieved with two Sylvania Oracle lamps per treatment tank with 150W HIS-TD Coral-Arc bulbs suspended 85 cm above each treatment tank (Sylvania, Sydney). The light intensities per lamp and at different positions in the tanks were measured with an Extech EA33 dome-sensor light meter (Triosmartcal, Australia), and lamps as well as sponge sets per tank were additionally systematically rotated. The overall mean light intensity was 7030 cd, and intensities were not significantly different between the experimental tanks (n = 5 measurements per tank). Due to heat generated by the lamps, the water temperature followed a diurnal cycle with the same rhythm and amplitude of ∼ 1°C in all tanks, simulating a natural temperature oscillation as on the reef.

### Monitoring Carbonate System Parameters

Temperature in the treatments was recorded in 10 min intervals with Starmon Mini high-resolution loggers (Star Oddi, Iceland; accuracy ±0.02°C). Salinity (PSS scale) and pH (NBS scale; for monitoring purposes only) were measured daily with a SevenGo DUO meter (Mettler-Toledo, Switzerland) equipped with an InLab 738-ISM conductivity probe and an InLab Expert Pro-ISM pH probe (both calibrated daily with NIST certified buffer solutions). Water samples were taken at the start, the 3^rd^, 6^th^ and 10^th^ day of the experiment. Sampled water was sterile-filtered with 0.2 µm PES filters. Samples for DIC and Total Alkalinity (TA) were treated with 0.02 vol % saturated HgCl_2_ solution to arrest biological activity while samples for nutrients were left untreated. The relative order and timing of all sampling procedures between 9∶30 and 10∶30 h was kept constant in order to minimise influence of daily fluctuations in temperature, pH, and sponge-dinoflagellate biorhythms. During this time window, pH and T were closest to their mean values (see below). Nitrate, nitrite, phosphate, and silicate were measured photometrically (U-2000, Hitachi, Japan) with precision levels of ±0.5, ±0.02, ±0.05, and 1.1 µmol/l; ammonium was quantified fluorometrically (SFM 25, Kontron Instruments, Germany) with a precision of ±0.08 µmol/l. TA was determined in duplicate, using potentiometric open-cell titration. Seawater was weighed (1416B MP8-1, Sartorius, Germany) and titrated with 0.005 N hydrochloric acid in an automatic titrator (Titrando 808, Metrohm, Germany); the average precision between duplicate water samples was ≤4 µmol/kg. DIC was measured photochemically using an automated segmented flow analyser (QuAAtro, Bran+Luebbe, USA) equipped with an autosampler (±5 µmol/kg precision). Both, TA and DIC were calibrated with certified seawater reference material (Dickson standard). The carbonate system was computed from the measured temperature, salinity, TA and DIC concentrations using the CO2SYS program (EXCEL macro v. 1.02 in default settings). All nutrient levels were very low with values near or below precision and detection levels, rendering consideration for correcting the carbonate system calculations unnecessary. As a cross-check, calculated (via DIC and TA) and directly measured pH values were compared and showed a highly significant correlation and no outliers (pH_(total scale)_ = 0.9176 pH_(NBS)_ +0.5453; r^2^ = 0.99; p<0.0001). This regression was used to convert measured pH values to total scale. Numerical data of the experimental settings and selected calculated carbonate system parameters are given in Table 1 and are illustrated figure 3.

### Assessment of Bioerosion Rates

Bioerosion rates were determined gravimetrically by applying the buoyant weight method [24] at the beginning and the end of the experiment after 10 days of exposure. This method allows an accurate determination of substrate weight loss, while the sponge tissue (<2% of the dry weight) with a density much closer to that of seawater does not carry any relevant weight for our purpose. High precision was assured by placing the balance (CPA324S, Sartorius, Germany) on a weighing chamber in a tank with experimental outflow water, using a harness with tungsten wire, keeping harness and reference weight underwater all the time, weighing the reference weight every 5 samples to enable drift control, and by using the animal weighing mode to reduce bias by vibrations (average of 100 measurements in 20 s). Since bioerosion is commonly quantified as removed substrate mass per unit surface area and time, bioerosion rates were converted to the commonly used unit kg m^−2^ yr^−1^ by relating the weight difference to the surface area of the sponge. After the healing process, the sponge-bearing cores had two surface areas to consider, the upper circle of original surface and the healed surface around the sides of the core. As the latter is in right angle to the upper surface and will thus have a lesser influence on the bioerosion, we included only half of the lateral surface in the reference unit in order not to underestimate bioerosion rates.

### Quantification of Sponge Tissue and Penetration Depth

After the experiment, the amount of final sponge tissue and the mean penetration depth were determined per sponge-bearing core (n = 32 per treatment). The cores were soaked in freshwater for 24 h to remove the salt, rinsed with deionised water, and dried at 110°C for 60 h, before determining the dry weight (CPA324 S, Sartorius, Germany). Obtained values served as a validation for the buoyant weight method and the calculated values differed from the directly measured dry weight by only 0.06±0.05%. The sponge tissue was then removed with ∼ 5% hydrogen peroxide and the weighing procedure was repeated for determining the weight difference corresponding to the sponge biomass. The mean penetration depth of *C. orientalis* was quantified with a digital calliper from the two deepest and two shallowest penetration depths of each core.

### Statistical Analyses

Linear regression models were computed with SigmaPlot (v. 12). Normality tests, One-way ANOVA, and the Kruskal-Wallis analysis were carried out with PAST (v. 2.03). Two-way ANOVA of PAM data was undertaken with the software R (v. 2.13).

## Results and Discussion

**Figure 4:**
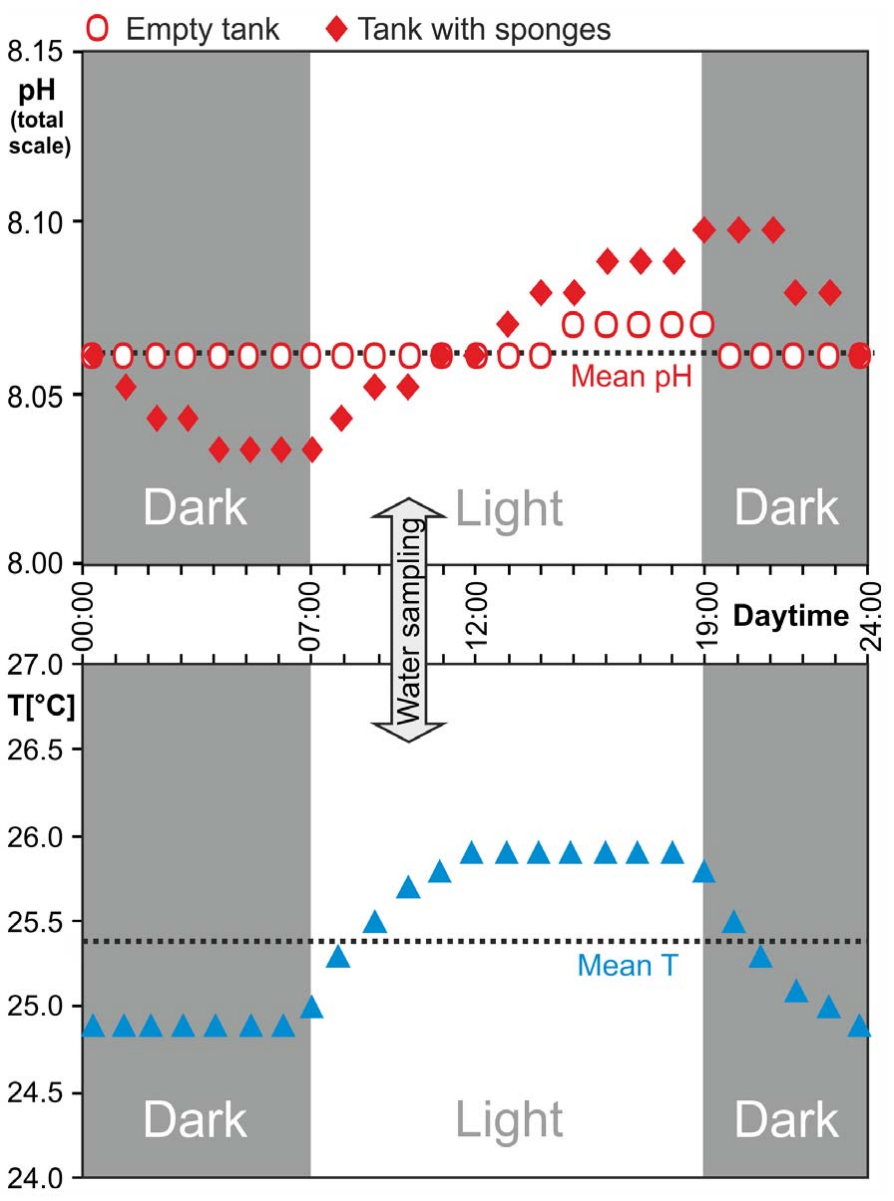
Diurnal pH and temperature oscillations. Biologically induced pH fluctuation (increase during photosynthesis; decrease as result of respiration) in the present-day *p*CO_2_ treatment tank (393 µatm) with (diamonds) and without sponges (circles), showing the causal relationship with the illumination phase (top); temperature fluctuation in the same tank affected by heat radiated off the metal halide lamps (bottom, triangles).

Before testing a possible pH dependency of sponge bioerosion, we assessed biologically-driven daily pH fluctuations in the treatment tanks as evidenced during a 24 h series of measurements logged both with and without sponge replicates in place (Fig. 4). Despite the flow rate of ∼30 l/h, a pH oscillation of 0.07 points was determined at present-day *p*CO_2_ when sponges were in the tank. The rise in pH coincided with the beginning of the 12 h irradiance period (simulated daylight), and values declined again after lights were turned off. This signal reflects the uptake of CO_2_ (and linked rise of pH) during active photosynthesis of the symbionts in the sponge tissue. This flux was higher than the simultaneous generation of CO_2_ from the sponge respiration, resulting in net photosynthesis during daytime. In contrast, during the following dark phase only respiration was taking place, both by the sponge and its photosymbionts, and led to a decrease in pH. In comparison, the photosynthetic activity of phytoplankton and some early algal turfs in the treatment tanks amounted to a change of only 0.01 pH points. The temperature in all treatment tanks also followed a synchronous light-dependent rhythm due to the warming by the lamps, thereby simulating daily temperature fluctuation. The carbonate saturation states for aragonite and calcite never became undersaturated (Ω <1), neither in the highest experimental *p*CO_2_ nor when considering the diurnal pH and temperature fluctuations. Nevertheless, a relevant abiotic dissolution or microbial bioerosion of the coral substrate was ruled out by including clean dead coral cores of similar size and from the same source as controls – none of these lost weight, despite the larger exposed surface in the sponge-free cores (Table 1). Estimates of proportional biomass [25] additionally indicate that microbial bioerosion by phototrophic or chemotrophic euendoliths in the sponge cores is negligible. We furthermore checked mean penetration depth and sponge tissue weight per sponge-bearing core after the experiment (Table 1), which did not vary significantly between treatments and confirmed that our data were not biased by sponge biomass or tissue shrinkage, so that the change in buoyant weight recorded in our experiment can be addressed with confidence to the chemical and mechanical bioerosion activity of *Cliona orientalis*.

**Figure 5:**
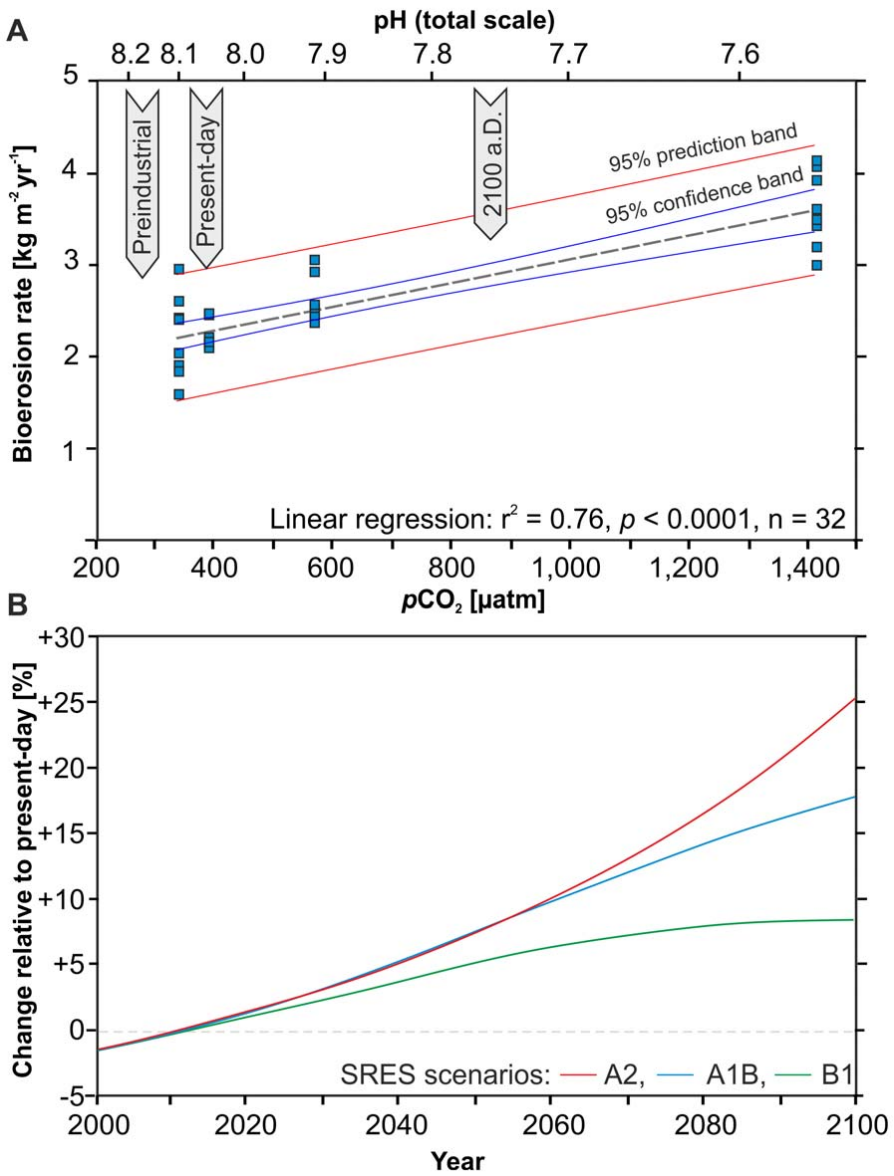
Increasing sponge bioerosion as a function of increasing *p*CO_2_. (A) Weight loss per replicate set translated to bioerosion rates for the four *p*CO_2_ treatments. The linear regression of the 32 replicates (8 per treatment) is highly significant (r^2^ = 0.76; *p*<0.0001). (B) Projected percent increase in sponge bioerosion relative to the present-day level, calculated for the BERN-CC model based on the SRES A2 (red), A1B (blue), and B1 (green) emission scenarios.

Sponge bioerosion rates reached a mean of 2.23±0.15 kg m^−2^ yr^−1^ in the present-day treatment (Table 1). Bioerosion rates significantly increased with rising *p*CO_2_ (Fig. 5A, Table 2). At moderately elevated *p*CO_2_ the mean bioerosion rate was 2.60±0.25 kg m^−2^ yr^−1^, which corresponds to a 17% increase relative to the present-day rate. At strongly elevated *p*CO_2_, bioerosion was further enhanced, attaining a mean rate of 3.59±0.40 kg m^−2^ yr^−1^ and representing a 61% change compared to the present-day value. This increase in bioerosion rate reflects the enhanced efficiency of the sponges’ bioerosion process as a result of the lowered environmental pH, causing a shallower gradient between the environment and the etching site. The sponge apparently ‘takes advantage’ of the facilitated dissolution in the more acidic environment, as opposed to keeping bioerosion rates constant and only lowering the metabolic cost. In contrast to this distinct trend, a decrease in bioerosion rate of 2.22±0.45 kg m^−2^ yr^−1^ in the slightly lowered *p*CO_2_ level was less than 1% lower and thus not significantly different compared to the present-day treatment (Table 2). The physiological interpretation for our findings in the lowered *p*CO_2_ is that the sponge is partly able to compensate for the less favourable conditions (hindered dissolution in more alkaline conditions), possibly at the cost of increasing the metabolic rate. The overall linear regression of bioerosion rate versus *p*CO_2_ is highly significant (r^2^ = 0.76; *p*<0.0001) and clearly supports the initial hypothesis that sponge bioerosion can be expected to accelerate with progressing OA. Based on the linear regression, the relationship between *p*CO_2_ [µatm] and *C. orientalis* bioerosion rates [kg m^−2^ yr^−1^] can be formulated as in Eq. 1, and the respective relationship converted to changes in pH in Eq. 2.
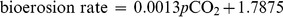
(1)

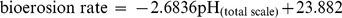
(2)


Keeping the limitation in ecological relevance inherent to short-term lab experiments in mind, this relationship translates to a predicted 25.4% increase in sponge bioerosion by the end of this century, following the BERN-CC reference model based on the SRES A2 emission scenario that corresponds to a predicted *p*CO_2_ level of 836 µatm by the year 2100 [26]. The most optimistic SRES B1 model with a predicted 2100 *p*CO_2_ of only 540 µatm would result in an 8.6% increase and the intermediate SRES A1B model with a 2100 *p*CO_2_ of 703 µatm equates to a potential 17.7% increase in sponge bioerosion (Fig. 5B). A similar range of predictions can be made when applying the new Representative Concentration Pathways (RCPs) [27] with an 8.6%, 15.8%, and 30.9% increase for the RCP 4.5, 6, and 8.5 scenarios, respectively.

**Table 2:** Results (*p* values) from the pairwise comparison of bioerosion rates in the four *p*CO_2_ treatments (Mann-Whitney test; Bonferroni corrected *p* values in lower left triangle of matrix) performed after Kruskal-Wallis analysis (H = 21.25; Hc = 21.25; *p*<0.0001; n = 8 per treatment) and rejection of normal distribution for the present-day (393 µatm) and the elevated treatment (571 µatm) via Shapiro-Wilk test.

	below present *p*CO_2_339 µatm	present-day *p*CO_2_393 µatm	elevated *p*CO_2_571 µatm	strongly elevated *p*CO_2_1410 µatm
below present *p*CO_2_ 339 µatm	–	0.7132	0.0831	0.0009*
present-day *p*CO_2_ 393 µatm	1.0000	–	0.0063*	0.0009*
elevated *p*CO_2_ 571 µatm	0.4987	0.0379*	–	0.0014*
strongly elevated *p*CO_2_ 1410 µatm	0.0056*	0.0056*	0.0082*	–

* significant difference.

Due to the important role of bioeroding sponges, and of ‘*C. viridis* complex’ species in particular, this finding suggests severe consequences for coral reef health. Coral reef calcification and bioerosion are antagonistic processes in a dynamic balance [28], [29]. This balance will become seriously strained when bioerosion is accelerated by OA, while at the same time, coral net calcification rates are declining [4]–[8]. This situation will push the carbonate budget towards negative values, and on some reefs negative carbonate budgets have already been recognised as result of intensive sponge bioerosion [16], [17].

Pioneer experimental evidence for an increase of bioerosion rates generated by specific bioeroders due to seawater acidification was provided for euendolithic microborers. Biosphere 2 experiments showed that particularly the dominant microboring chlorophyte *Ostreobium quekettii* grows faster under elevated *p*CO_2_ (750 µatm) [10]. However, in contrast to endolithic algae which occasionally even support stressed calcifiers [30], bioeroding sponges are always in antagonism to calcifiers, and the species we worked with is known to often overwhelm and kill live corals [18], [21]. Several mesocosm experiments and field studies demonstrated an increase of total dissolution – including bioerosion, but rarely addressed as such – partly leading to a net loss of carbonate [31]. Coral reefs in the eastern tropical Pacific, where cool, CO_2_-rich upwelling water masses lead to naturally low pH and saturation states, are poorly cemented and prone to intense bioerosion [17], serving as a model for coral reef development in a high-CO_2_ world [9]. Lowest mean pH_(seawater scale)_ values of 7.88 and a corresponding Ω_Ar_ of 2.49 were reported from Galápagos [9]. Hence at least part of pH conditions predicted by OA scenarios is already experienced by tropical reef corals and bioeroding sponges at present time. This may apply not only for the eastern Pacific but for many shallow coral reefs with relative long water residence times, as a result of carbon fluxes related to calcification and the remineralisation of organic matter [32]. At the GBR for instance, spatial and temporal pH fluctuations are in the magnitude of 0.4 pH units [33]. Hence, the three lower *p*CO_2_ treatments of the present experiment were within the range of natural fluctuations currently experienced on some coral reefs, whereas the strongly elevated treatment looks far into the future and may never be reached.

Intriguingly, another important factor in climate change – global warming – may partly counteract the development caused by OA. Rising temperatures reduce the physicochemical dissolution capacity of calcium carbonate in seawater [34] and could also slow down chemical bioerosion. However, within tolerance limits of physiological processes, i.e. chemical reactions can be accelerated by elevated temperature, and interaction of *p*CO_2_ and temperature may have complex effects as has been demonstrated with respect to coral calcification rates [35], [36]. This observation calls for multifactorial experiments that consider both, the isolated as well as concerted effects of *p*CO_2_ and temperature on sponge bioerosion and other bioerosion processes. And, as an indispensable step, the impact of climate change on bioerosion needs to be addressed in long-term in-situ experiments. Ultimately, these data will convey critical insights into global trends of biologically caused decalcification and the possible threat of increasing bioerosion on the balance between skeletal growth and bioerosion on tropical coral reefs.
